# Biologicals and Fetal Cell Therapy for Wound and Scar Management

**DOI:** 10.5402/2011/549870

**Published:** 2011-05-18

**Authors:** Nathalie Hirt-Burri, Albert-Adrien Ramelet, Wassim Raffoul, Anthony de Buys Roessingh, Corinne Scaletta, Dominique Pioletti, Lee Ann Applegate

**Affiliations:** ^1^Cellular Therapy Unit, Department of Musculoskeletal Medicine, University Hospital of Lausanne, CHUV/UNIL, PAV 03, 1011 Lausanne, Switzerland; ^2^Office of Dermatology and Angiology, Place Benjamin Constant 2, 1005 Lausanne, Switzerland; ^3^Department of Plastic and Reconstructive Surgery, University Hospital of Lausanne, CHUV/UNIL, BH 10, 1011 Lausanne, Switzerland; ^4^Department of Pediatric Surgery, University Hospital of Lausanne, CHUV/UNIL, BH 10, 1011 Lausanne, Switzerland; ^5^Biomechanical Orthopedics Laboratory, Swiss Federal Institute of Technology, EPFL, 1015 Lausanne, Switzerland

## Abstract

Few biopharmaceutical preparations developed from biologicals are available for tissue regeneration and scar management. When developing biological treatments with cellular therapy, selection of cell types and establishment of consistent cell banks are crucial steps in whole-cell bioprocessing. Various cell types have been used in treatment of wounds to reduce scar to date including autolog and allogenic skin cells, platelets, placenta, and amniotic extracts. Experience with fetal cells show that they may provide an interesting cell choice due to facility of outscaling and known properties for wound healing without scar. Differential gene profiling has helped to point to potential indicators of repair which include cell adhesion, extracellular matrix, cytokines, growth factors, and development. Safety has been evidenced in Phase I and II clinical fetal cell use for burn and wound treatments with different cell delivery systems. We present herein that fetal cells present technical and therapeutic advantages compared to other cell types for effective cell-based therapy for wound and scar management.

## 1. Introduction

Cell-based therapies are penetrating gently into routine medical care and especially for wound management of skin. They offer the promise of repairing and/or replacing damaged tissue and restoring lost functionality, because ideally, they provide all of the factors necessary for wound healing. Several cell types and tissues have been proposed as starting material including autologous cells, adult stem cells including those derived from bone marrow, and adipose tissue, fetal cells, embryonic stem cells, platelets, and tissues from placental and amniotic fluid. These cell types are used for biological preparations in processing vaccines and medicinal, veterinary, and tissue engineering products [1–41]. As the literature and information is vast on cell-based therapies, this paper will concentrate on fetal cells as the choice in wound and scar management. Firstly, we will define differences between stem, and mesenchymal and fetal cells, as the literature is confusing with these terminologies, followed by a short review of fetal wound healing and associated processes. Importantly, cell choice and the technical specifications to outscale, stability, safety, and delivery are the major hurdles for development of biologicals for better wound treatments and scar management. Fetal skin cells present biological, technical, and therapeutic advantages lending towards possible routine cellular-based therapy for wound and scar management. All of these aspects will be addressed in the description of how dedicated fetal skin cell banks can be developed, potential delivery systems, and cellular mechanisms of repair with gene profile differences between fetal and young skin cells to illustrate biological families implicated in wound healing. Finally, the capacity of this cell type in wound and scar management is illustrated and summarized from Phase I and II clinical safety studies in humans.

### 1.1. Cellular Sources as Therapeutic Agents: Terminology Clarification, Technical Requirements, and Cell Banking

**Figure 1:**
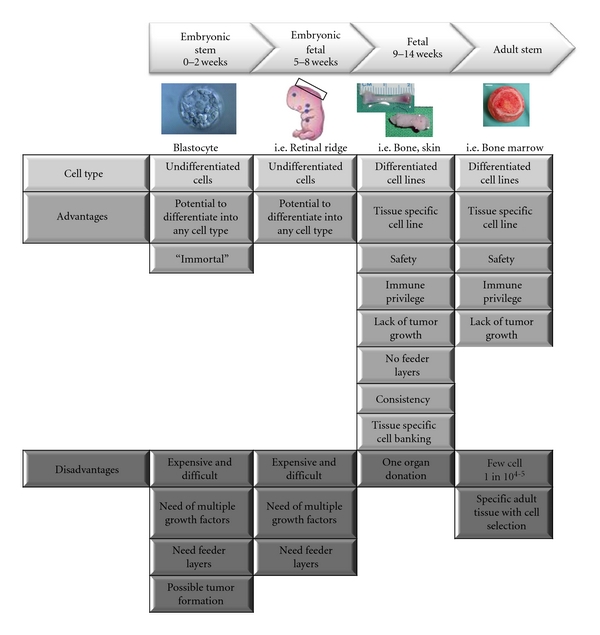
Cellular source and development stage with advantages and disadvantages.

There is some confusion between the terminology and potential of embryonic, fetal, and adult stem cells. These cells are referred to in the literature as embryonic stem cells, fetal cells, and mesenchymal stem cells, respectively. However, more frequently, all of these cell types are referred to as simply stem cells, neglecting all of the legal and technical aspects associated with each specific cell type. To illustrate these differences, Figure 1 lists the major cell sources used in developing therapeutic applications showing that some cell choices are more adaptable to cellular therapy in patients. This adaptability is highly associated with technical facility of expanding and selecting cell populations needed. Tissue choices from animal and human at all ages of development can be evaluated with advantages and disadvantages for each final cell type (Figure 1). In legal aspects, the term “embryo” denotes the earliest stages following fertilization of an ovum by a sperm. Zygote would include early stage cleavage embryos produced by cell division up to 50–60 cell stage (each cell which is a blastomere) and the blastocyte for the 60 cell stage to the point of implantation at about 2 weeks after-fertilization. “Embryonic stem cells” (ES) are developed from preimplantation embryos from the inner-cell mass before the first 2 weeks of development. These cells are frequently obtained from extra embryos developed by “*in vitro*” fertilization techniques to aid couples for fertility purposes. Because these particular cells have created quite an ethical debate, other researchers have begun using “fetal embryonic cells” derived from voluntary interruption of pregnancy between 5 and 8 weeks. Cell lines are normally developed from the genital ridge of the fetus. As this tissue is considered as an organ donation in most countries, it bypasses the major problems that have been raised by embryonic stem cells. Most fetal cell research is developed from specific tissues such as skin, muscle bone, or cartilage at the latter end of the first trimester (11–14 weeks) following voluntary interruption of pregnancies. Cell lines at this stage are tissue specific, and therefore, “fetal cells” are differentiated with specific functions. Fetal cells are derived from a legal organ donation when the mother donor ensures informed consent, provided that the tissue is a donation and not paid for and that there is no change in timing or method of pregnancy interruption for the sake of research. As fetal cells are from organ donations and under transplantation medicine, they are also routinely included in the terminology as adult stem cells.

Adult stem cells can be established from most tissues of the body, and the most frequently isolated ones come from bone marrow and adipose tissues of young and adult individuals and are referred to as “mesenchymal stem cells” (MSCs). 

Each of the cell types mentioned in Figure 1 have different technical requirements for producing appropriate cells that could be used as therapeutic agents.

Embryonic stem cells that are isolated from early-stage embryo present the particularity of being pluripotent and have an advantage over those cells from adult mesenchymal stem cells, which can differentiate only into a restricted number of cell lineages. However, cultures of these stem cell types are technically very demanding, because the amount of tissue to begin is very low for embryonic stem cells and the isolation of mesenchymal stem cells from the tissue mass is difficult (only 1 stem cell for every 10^4-5^ cells in total adult tissue). Maintenance and expansion of stem cells in an undifferentiated state require the addition of many specific growth factors [28, 29], and so far, culture of embryonic stem cells and some mesenchymal stem cells are not possible without feeder layers which is in some part responsible for the inconsistent colony cell growth [29]. The necessity to use many exogenous growth factors as well as feeder layers to differentiate into specific cellular lineages are limiting factors for the scaleup of stem cell cultures for clinical applications. There are other major issues with these stem cell types for security as the cells can dedifferentiate once placed into an *in vivo* environment and even develop into tumors. Many techniques involving cell cloning or encapsulation would be necessary for assuring delivery of correct cell populations.

Unlike stem cells, fetal cells are differentiated cells with high expansion, regeneration, and low immunogenic properties [10, 12]. As the fetal cells are already differentiated and do not need to be directed or altered, the vast number of additional growth factors normally necessary are not needed for cell culture and expansion, and these cells are not known to dedifferentiate once placed into the *in vivo *environment [7–9, 12]. 

Establishment of cell banks is a crucial step in the process of many vaccines, medicinal products, or tissue-engineering products, and therefore, the choice of cell type is extremely important for technical and security reasons. A “cell bank” is the stocked product of consistent cell cultures that are frozen into small vials that withstand long-term freezing in liquid nitrogen (−165°C). The initial cell bank is frequently termed the master cell bank (MCB) from which each vial can derive a working cell bank (WCB). Whole cell bioprocessing and adaptable procedures to good manufacturing processes (GMPs) make it possible to develop extensive MCB and WCB to facilitate thorough testing of the cells. Once MCBs are accomplished, WCB can be produced to establish individual batches of treatments for high numbers of patients. Further, these cell banks can be tested completely for safety regarding sterility, pathogens and adventitious agents, and tumorigenicity. 

Historically, fetal cells have been used in cell banking procedures for medicinal products for many years already. Already in the 1930s, medical doctors and scientists have used tissue from voluntary pregnancy interruptions not only for understanding cell biology but also an important entity in the development of vaccines by using defined tissue-derived cell lines. The Nobel Prize for Medicine in 1954 was awarded to American immunologists who developed the polio vaccine based on cultures of human fetal cells. Since this time, many other necessary vaccines (rubella, chicken pox, hepatitis A, etc.) have been developed with the use of fetal cell lines including two primary human diploid cell lines which were originally prepared in the 1960s. The first cell line, WI-38 (Wistar Institute 38), was developed by Leonard Hayfleck in 1964 from fetal tissue from a voluntary pregnancy interruption and later given the ATCC (American Type Culture Collection) number of CCL-75. This cell line was used for the historical production of vaccine RA 27/3 against Rubella.

### 1.2. Fetal Wound Healing

Considerable interest and research have been dedicated to the understanding of wound healing and the associated process. Whereas adult cutaneous wounds heal more slowly and with scar formation to restore tissue integrity, fetal skin, *in utero*, is observed to have rapid and scarless tissue repair characterized by regeneration of an organized dermis with normal appendages and by a relative lack of inflammation [42–45]. Fundamental differences between fetal and adult skin and the fetal and adult skin wound environment may be important in inducing efficient tissue repair. 

 Molecular analysis of wound healing has largely been devoted to cytokines and most particularly those of the transforming growth factor (TGF) family and their role in manipulating cutaneous wound healing and scar formation [46]. It has been suggested that scarless wound healing in fetal skin at early gestation is a result of the unique cytokine or growth factor profile [3, 45]. Of these, transforming growth factor-beta (TGF-*β*) has been most widely studied as it is implicated in the transition between scarless healing and repair with scar formation [47].

Three highly homologous TGF-*β* isoforms are known in humans: *β*1, *β*2, and *β*3 [46]. Each form has been found by immunohistochemistry in unwounded fetal skin. However, low levels of TGF-*β*1 and high levels of TGF-*β*3 are expressed at gestational ages associated with scarless repair [3, 45]. 

Exogenous application of TGF-*β*1 to normally scarless fetal wounds resulted in scar formation with an adult-like inflammatory response observed [47]. The profibrotic nature of TGF-*β*1, and possibly TGF-*β*2, was confirmed in wounds of adult rats, since neutralizing TGF-*β*1 and *β*2 with antibodies partially reduced the amount of scarring [48]. However, antifibrotic properties can be seen with the isoform TGF-*β*3 as injection, or application of this isoform showed reduced scarring and inflammation in adult wounds [48, 49]. When using a rabbit hypertrophic scar model, TGF-*β*3 was confirmed to show increased properties in wound healing but not scar reduction [50]. It has been suggested that the relative proportion of TGF-*β* isoforms, and not the absolute concentration of any one isoform determines the wound repair outcome [3, 45–47]. 

TGF-*β*2 has been shown to be significantly higher in fetal skin dermal fibroblasts than in foreskin tissue fibroblasts but no major differences with TGF-*β*1 and TGF-*β*3 [11]. When looking at expression of the three isoforms in five different fetal skin cell lines, it was observed that TGF-*β*1 gene expression is much higher than that of TGF-*β*3. Importantly, variability between fetal donors for the TGF-*β* isoforms was very small which is generally the opposite for that of skin from young and old donors. Importance has been given to the TGF-*β*3 isoform by a company in England, Renovo Ltd., who have used human recombinant TGF-*β*3 (Juvista) in clinical trials showing 70% response rate for scar reduction to date. 

However, TGF-*β*1 and *β*2 neutralizing antibodies do not entirely prevent scarring in the adult, and other studies question the efficacy of TGF-*β*3 in wound healing [50]. 

More recently, inhibition of TGFbRII-mediated signaling was demonstrated with a gene-therapy approach in a rabbit hypertrophic scarring model showing some reduction biologically. Lack of complete reduction could either be due to the technical difficulties suggested by the authors for low transduction efficiency, or it may suggest that factors other than TGF-*β* may also be important in scarless repair. As TGF-*β*'s particular importance in wound healing is also due to their ability to modulate ECM formation, two genes have previously been shown in wound healing in fetal skin (laminin b1, LAMb1) and in hypertrophic scar (dermatopontin, DPT) [51]. Dermatopontin and laminins have important roles in cell-matrix interactions and matrix assembly, and dermatopontin has been shown to be decreased in hypertrophic scar and systemic sclerosis skin fibroblasts [52]. 

As wound healing is very complex, there are certainly many other molecules within the TGF-*β* superfamily which could have a role. For instance, bone morphogenic protein (BMP) family of genes and their receptors are among those in the TGF-*β* superfamily genes and have also been strongly associated with cutaneous wound healing and scarless wound healing in the fetus. Overexpression of BMP-6 was shown to delay re-epitheliazation and promote scar formation in a transgenic mouse model [53]. BMP-6 is important for maintaining skin homeostasis and is 3.8 times higher expressed in fetal dermal fibroblasts than in newborn foreskin fibroblasts. More recent studies have shown evidence for the importance of angiogenesis and nerve involvement in wound repair [54, 55]. Pleiotrophin (PTN), a cytokine inducing heparin-binding/differentiation, is certain to have a major role in angiogenesis in wound healing. Midkine (MDK) and PTN, which have 50% amino acid sequence identity and striking domain homology, are the two members of the *Ptn/Mdk* developmental gene family [55]. Interestingly, PTN has been recently shown to induce functional neovasculature *in vivo* [55]. As fetal tissue heals with no inflammatory response, lower MDK and PTN expression is perhaps preferable, as they could have an important role in angiogenesis.

In the past, gene profiling differences have been shown for fetal cells compared to adult cells [9], and therefore, it was also of interest to determine gene expression alterations in banked fetal dermal skin cells (used in tissue engineering for burns and wounds to date) and young dermal skin cells that have been banked in the same manner to have a listing of potential gene families from fetal banked cells to compare to young skin cells and other gene profiling studies. Young skin was, therefore, obtained from behind the ears of a 12 year old boy (12 yOS) following a surgical ear alignment and with the approval of the Hospital Ethics Committee to use this discarded material for research with parent and child oral and written approval. To identify differentially expressed genes in banked fetal dermal skin cells, we used cDNA microarray containing approximately 12,500 sequences (U95A human genome chip, Affymetrix UK, High Wycombe). Three arrays were hybridized for banked fetal skin cells (14 wFS) and banked young skin cells each (12 yOS). Briefly, RNA was isolated from cultured cells at passage one for each of the three cell banks. The statistical analyses were performed with a classical analysis of variance (ANOVA) followed by the global error assessment (GEA). Genes were selected by this method at *α* level 0.001. Significant genes, with at least a 1.5 fold change, were classified according to the gene ontology following the criteria of the DAVID (database for annotation, visualization, and integrated discovery) as described previously [9]. 

**Figure 2:**
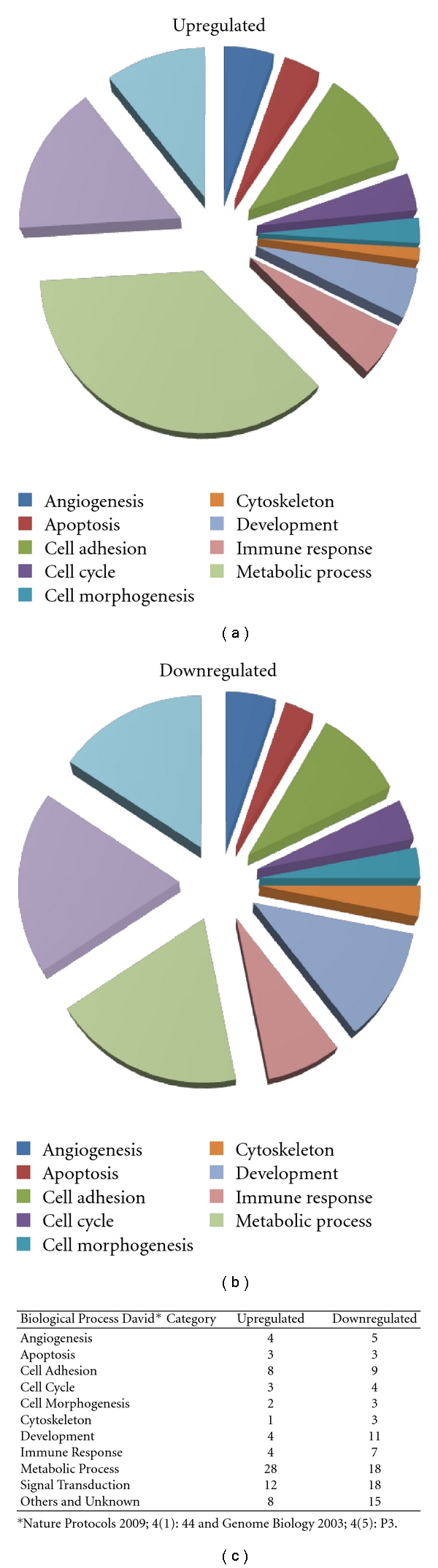
Gene profiling and biological processes of fetal versus young skin cells. A cDNA microarray containing approximately 12,500 sequences (U95A human genome chip, Affymetrix UK, High Wycombe) was used to identify differentially expressed genes in banked fetal dermal skin cells compared to young skin cells. Three arrays were hybridised for each separate cell bank, and gene ontology of important differentially expressed genes was annotated following the criteria of the DAVID database (http://david.abcc.ncifcrf.gov/) for annotation, visualization, and integrated discovery. Biological processes of gene ontology is reported in the pie graphs for both up- and down-regulated genes (category for their best representation).

When comparing banked fetal dermal skin cells to banked young fibroblasts with our conditions, 167 genes changed by 1.5 fold or more. Between those genes, 74 were upregulated in fetal cells and 93 were downregulated (Table 1). Gene ontology of important differentially expressed genes was annotated following the criteria of the DAVID database (http://david.abcc.ncifcrf.gov/) for annotation, visualization, and integrated discovery) [56, 57]. As many of the genes analyzed could be involved in multiple biological processes, they have been placed in a category for their best representation (Figure 2). 

Although individual growth factors (TGF-*β*2, TGF-*β*3, IL-10, and PDGF) have been shown in the clinic to help in different aspects of overall wound healing, it is indeed a very complex process [3, 45]. Most likely, many factors taken together are necessary for complete wound closure which could indeed be offered by a cell-based therapy for which many factors could be different in fetal cells such as those found in Table 1.

Indeed, it has been shown that efficient repair could be obtained in 2nd and 3rd degree burns in children [7], acute wounds [8], and in chronic wounds [9] to date, and the whole-cell bioprocessing and age of the cells were very important aspects [10, 12].

## 2. Fetal Tissue Collection, Culture, and Cell Bank Requirements

One of the major challenges for assuring that more patients will benefit from cell-based therapies in the future will be the optimisation of the choice of cell type as well as their isolation and proliferation. The development of master cell banks from the cell choice provides a major advantage for the creation of a therapeutic biological agent. Careful selection of donors and extensive screening of both the donor and cultured cells avoids transmissible viral, fungal, or bacterial disease and, therefore, can provide a safe and secure utilization of cells for therapeutic purposes.

Fetal cells have the advantage of being an organ donation, and only very small biopsies are necessary for developing extensive, consistent master cell banks for many tissues. Thorough safety testing of mother donor for infectious diseases (status of the donor for HIV, HBV, and HCV) at the time of tissue donation and again 3 months after to ensure negative sero-conversion can be ensured. During the informed consent of the patient, an interview for overall health is recorded to evaluate eligibility into an organ donation program. For the tissue, information concerning the age of the fetus, date, time, and place of acquirement need to be obtained along with types and amounts of tissue received. The transfer containers can be labelled with the identification code to assure an anonymous organ donation. All culture products used for the transfer of tissue and cell culture need to be of clinical grade quality. Cell banking and testing criteria need to be accomplished under cGMP conditions such as required for human diploid cells used for vaccine production and for cell substrates used for biotechnological products. Testing routinely accomplished on cell banks includes tests for viruses, virus-like particles, mycoplasmas, fungi, yeasts, and bacteria with both *in vitro* and in vivo testing. Donor source is identified by isoenzyme testing, and *in vitro* testing of picornavirus, orthomyxovirus, pramyxovirus, herpesvirus, adenovirus, and reovirus is routinely accomplished with several control cell lines when the tissue is human source (Q-PCR for HepB, HepC, HIV-1, HIV-2, HTLV-1, HTLV-2, HHV-6, HHV-7, HHV-8, EBV, hCMV, SV40, and B19 parvovirus). *In vivo *virus testing using suckling mice, adult mice, guinea pigs and embryonated eggs is routinely employed.

It is important to note that until now, there have been no reported biopharmaceuticals derived from continuous cell cultures that have been implicated in the transmission of infectious agents to humans. Most sources of contamination are adventitious, which means that the contamination is introduced from an external source such as the culture products.

## 3. Cellular Delivery Systems: Collagen Matrix, Cream, and Hydrogel

Once safety can be assured, efficient cell presentation with biocompatible delivery systems can be assessed for specific tissues. For delivery systems, biocompatible biomaterials need to be available in order to provide an extracellular matrix environment for cell differentiation, delivery, and release. To be admitted for clinical use, it is more rapid to use approved medical device quality material. Cell and materials need to be tested together to assure not only biocompatibility but also their interactions, cellular stability, possible degraded by-products of combination, and degradation or absorption. Ease of applicability of the final product will be of importance for clinical use.

Preparations from biologicals, and particularly from live cells and their delivery to the patient are a major complication for treatment, and the shelf life is very limited with such preparations. Many products for skin repair have applied alternatives such as freeze-dried, frozen, and refrigerator stocking [18–23]. Stability and bioequivalence will have to meet the exigent and stringent technical aspects for development of therapeutic products. 

**Figure 3:**
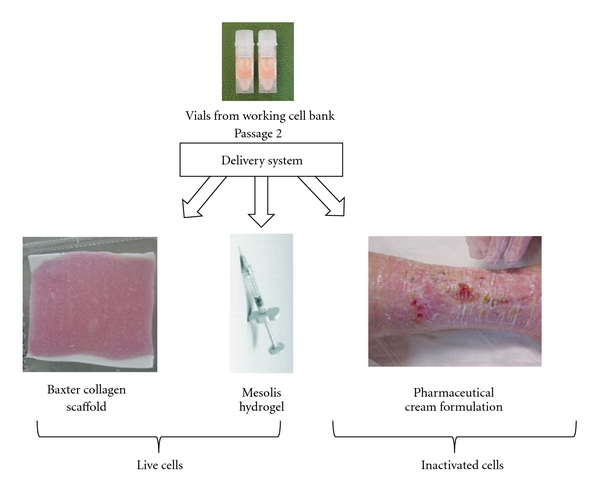
Cell delivery by “biological bandage”, hydrogel, and pharmaceutical cream formulation. Each identical ampoule of fetal skin cells (working cell bank) can be seeded directly into biocompatible collagen (Tissue Fleece, Baxter, Switzerland), hydrogels (Mesolis, Anties, Plan-les-Ouates, Switzerland), or into pharmaceutical cream formulations (oil-in-water emulsion base was prepared under GLP, Good Laboratory Practices) that assure biological stability.

Cellular therapies can be delivered as three-dimensional constructs (tissue-engineered live cell constructs) that can be placed topically over wound and scar surfaces or in solutions composed of creams or biogels. For large-surface wounds and areas that are difficult to apply overlying bandages, cream and biogel preparations permit an advantage for multiple daily applications with either live (hydrogel) or inactivated (cream) cells (Figure 3). Final preparations need to be consistent with equivalent “dosage” of cellular products delivered such as a cell density or by protein content.

## 4. Assessment of Fetal Cell Therapy in Wound and Scar Management

Management of wounds using fetal cell therapy have been intended for both acute and chronic wounds and for burns. In the first clinical studies reported using continuous fetal skin cell lines [7, 8], they were designed to prepare wound beds with fetal cells before autografting of patients who were programmed for secondary surgery for wound closure. This fetal cell preparative step was accomplished with the intention of having both a better “take” for the autograft and enhanced quality of treated skin. For pediatric burn patients, the pretreatment with fetal cells showed to be very efficient in wound closure to the point, where all 8 patients included in the study did not have to have the planned autografts for continued treatment [7, 8]. Treated skin showed complete closure rapidly with little hypertrophy of new skin and no retraction seen. 

**Figure 4:**
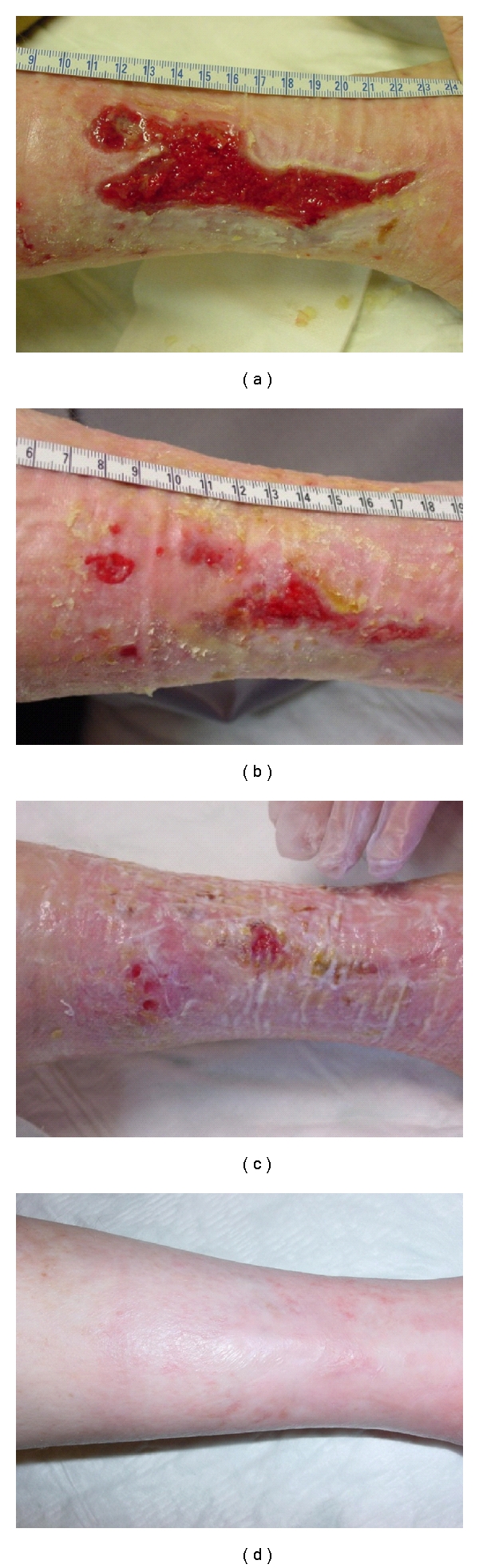
Fetal cell therapy of chronic wounds. Patient 1 (female 64 years old) with a history of painful postthrombotic ulcer for 14 years, consecutive to deep venous system, and short saphenous vein insufficiency (a). Evolution of the large, deep, and painful ulcer with applications of fetal skin constructs one time per week at 1 month (b) and two months (c). Following the majority of closure at 2 months, surrounding skin was treated with pharmaceutical cream preparation with inactivated fetal skin cells until full closure at 11 weeks (d). At one year followup, the patient shows skin that is still atrophic but no presence of scar tissue. “*Biological bandage*” *preparations*: fetal cells (seeding density of 5 × 10^3^ cells/cm^2^ with cells from the WCB at passages 3-4) were placed in culture media and seeded on the collagen sheet and placed into a 37°C incubator at 95% relative humidity and 10% CO_2_. Final products were employed for the patients in clinical Phase I and II studies for burns and wounds.* Cream preparation*: the cream was prepared under controlled, clean-room conditions in an automated pharmaceutical machine (Moltomat, Krieger AG, Basel, Switzerland). Its composition contained hydrogenated vegetable oil, glycerine, propylene glycol, cetearyl, ethyhexanoate, decyl oleate, ceteanyl alcohol, cetyl palmitate, glucose, ascorbyl palmitate, tocopheryl acetate, propylparaben, methylparaben, potassium chloride, magnesium chloride, sodium cetearyl sulphate, and simethicone. Samples were tested for quality control with respect to microbiological and physicochemical (viscosity, pH, conductivity, mass volume, colorimetry, and microscopy). Cell concentrations of 5 × 10^3^/mL were used on deteriorated skin of patients surrounding chronic ulcers and following wound closure of wound burn patients (Figure 5).

**Figure 5:**
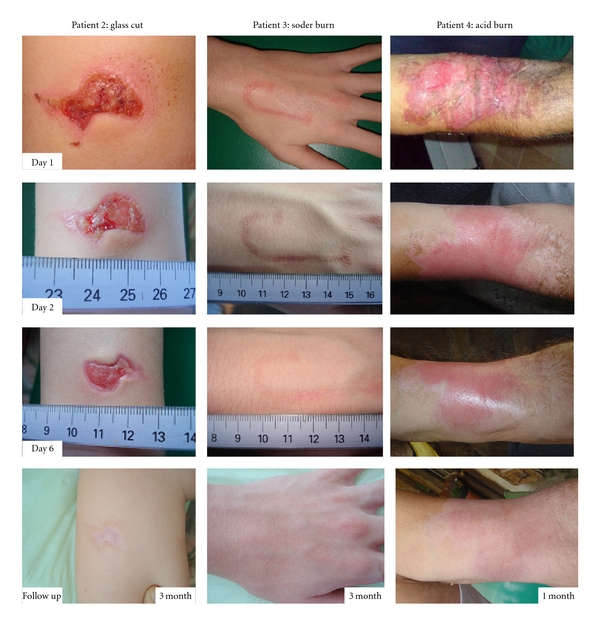
Patients with various forms of acute wounds (Patient 2) glass incision (Patient 3) soder burn, and (Patient 4) acid burn treated with fetal cell preparations as described for Figure 4. Evolution of wounds early in treatment with bandage changes every 2 days illustrates rapid wound repair with low associated inflammation.

In studies reported using fetal cells for chronic leg ulcers, patients were selected on the basis of a history of having refractory chronic leg ulcers, which did not heal using traditional therapies, such as compression (active and passive), hydrocolloids, and autografts [9]. Ulcers such as illustrated in Figure 4 (female patient 1, with a history of painful postthrombotic ulcer for 14 years, consecutive to deep venous system and short saphenous vein insufficiency) portray the use of several delivery systems of live and inactivated fetal skin cells. For this particular patient, previous autografts and different compression therapies including 4 layers bandages were not successful. Immediately following the first fetal skin construct, edema diminished, pain was relieved, and fibrin production elimination was evident. Applications of fetal skin constructs one time per week were capable of gradual but rapid closure of this large, deep, and painful ulcer. At 11 weeks, the larger portion of the ulcer was healed. Healed ulcer and surrounding skin was treated with pharmaceutical cream preparation with inactivated fetal skin cells until full closure. At one year followup, the patient shows skin that is still atrophic but with no presence of scar tissue (Figure 4). Although etiologies of ulcer patients are varied, similar observations were reported using fetal skin cells for treatment in 8 patients representing 13 ulcers included in the clinical safety studies [9]. Examples of rapid evolution of acute wounds and burns treated in the same manner with fetal cell preparations are portrayed in Figure 5. These patients were selected to represent the early repair processes noted and associated anti-inflammatory effects. Regardless of wound etiology (Patient 2 presented with an incision from a glass wound, Patient 3 with an intense soder burn, and Patient 4 with an acid burn), it can be generally observed that rapid epitheliazation of skin was initiated.

## 5. Discussion

Biologicals for wound and scar management have predominantly been developed with neonatal or young foreskin tissue cell culture to date (companies including, Smith and Nephew, XCELLentis, Organogenesis, Ortec, and DFB Pharmaceuticals) [13–15, 17, 18]. Differences of expression in wound healing gene families between fetal cells and foreskin cells used in biological preparations could be responsible for more efficient repair processes seen in the clinic and particularly for the rapidity in the treatment of acute wounds and burns [11]. Fetal skin represents the ideal paradigm of all tissue repair due to its inherent ability to repair through regeneration rather than scar. Even though fetal wound repair is a tightly regulated process involving many cellular mediators, the precise mechanisms of efficient wound healing without scar formation remain unknown despite the great increase in knowledge gained over the past decades. Chen et al. [58] have proposed that understanding the “blueprint of fetal skin repair” might allow the manipulation of adult wound healing in order to decrease scarring and fibrosis. There are indeed many genes that are significantly different in the “fetal skin blueprint” when compared to both younger and older skin that have been elucidated in previous work [9] and in this paper with banked fetal and young skin cells accomplished with the same cell banking techniques. Herein, we show that expression profiling of banked fetal cells compared to young skin has provided biological grouping of important gene families implicated in the mechanism of wound repair (such as cell adhesion, angiogenesis, and development; see pie graphs Figure 2, Table 1).

Indeed, individual growth factors (TGF-*β*2, TGF-*β*3, IL-10, and PDGF) have been shown in the clinic to help in different aspects of overall wound healing, but it is a very complex process. Most likely, many factors taken together are necessary for complete wound closure which could indeed be offered by a cell-based therapy for wound management. In making parallels with currently used medicines, it is now becoming more and more of a problem for single growth factors in wound and tissue repair. As milligram quantities are necessary for treatment of wounds such as that with platelet-derived growth factor (PDGF, Regranex), the long-term use is showing some safety concern. The use of this growth factor for the treatment of chronic leg ulcers has now been limited to short treatment regimens due to increased cancer in the patients (Ortho-McNeil-Janssen Pharmaceuticals; Important Drug Warning insert of medication). When only one growth factor or cytokine is used, the dosage accumulations are very high. Cellular therapies (or total cellular products) could provide the more correct balance for dosages of individual proteins or factors. The pmol/nmol quantities of individual proteins (when delivered all together are ~2-3 *μ*g) have been shown to be effective without the secondary effects on long term [7–9].

New biologicals may be of high interest if the safety and simplicity can be assured and if the overall cost can be limited. 

Regarding the use of the cells for preclinical trials, it will be particularly important to ensure consistency of growth of the cells and consistency of the harvest obtained. High consistency in fetal cell banking can be achieved due to the minimum requirements of fetal cell cultures. In contrast with MSC, fetal cells do not require feeder layers for growth nor growth factors for differentiation. Fetal cells show qualities required for the establishment of GMP cell banks to be used for medicinal and tissue-engineering products. The lifespan and the proliferation rate of the fetal cells can allow master cell banks of 100–300 ampoules containing 5–10 millions cells each from 2 cm^2^ of tissue at very early passaging (Passage 1 and 2). MCB and WCB have been prepared from fetal skin tissue in short periods of time compared to other primary cells [8, 10].

Regulation of final products and the simplicity will be mandatory milestones for biological development. Until 2007, Phase I and II safety studies with fetal cells were regulated in Switzerland (where the clinical trials were held) by the Department of Public Health (OFSP) under Transplantation Law (for living cell transplants) and by “hospital preparations in small quantities” by the state chemist (for inactivated cells). For comparison in the United States by the FDA, other cell types (i.e., foreskin) were regulated, in the majority, as medical devices or cosmetic products. New regulatory requirements issued in recent years (2007) will assure better safety, but cost will be largely affected. All cellular products must be in compliance with guidelines of good manufacturing practice (GMP) with respect to medicinal products and investigational medicinal products for human use. The European Union (EU) regulation on advanced therapy medicinal products (ATMPs) was adopted in all European Member States on Dec. 30, 2008, and the FDA recently also proposed regulations on human cells, tissues, as well as for cellular and tissue-based products. The main scope of the regulations is to establish clear classification criteria for many new cell-based medicinal products. For the EU, it makes reference to the 2004/23/EC directive on donation, procurement, and testing of human cells and tissues and also with directive 2002/98/EC on human blood and blood components. All together, these directives dictate that human cells have to be in compliance with the quality requirements therein described and that all ATMP have to be prepared under GMP conditions. Key elements including identity, purity, sterility, stability, safety, and efficacy are recommended for cellular-based products. In all, these new regulations impose strict criteria for the production and the environment used for the production of cell-based products to be used in clinical trials and treatments [59–69]. Evolution of advanced cellular therapeutics worldwide and how they are regulated will have a major impact on availability to patients. Clear regulatory affairs of cellular use, whether they are delivered living or inactivated, will be necessary to help researchers and clinicians in future therapies.

Cell therapies and tissue engineering are beginning to show great promise in wound and scar management. The cell choice is, therefore, an important factor for simplifying the overall technique and bringing therapy rapidly to the patient. 

Thus, fetal cells with their high expansion, simple culture conditions (do not require feeder layers or extensive growth factors for expansion which is a major reason for their consistency in scaling out), and low immunogenicity properties [10, 12] are ideal conditions for whole-cell bioprocessing destined for cell therapy, tissue-engineering, and medicinal products. Additionally, they have already been used in safety clinical Phase I and II studies showing rapid and efficient tissue repair with minimal scarring [7–9, 12]. 

Delivery systems to afford better stocking and stability will be important milestones for biological products, and topical preparations that show biological activity would be a great benefit. Overall, the advantage of cellular preparations is that there is no need for a chemical “active ingredient” for wound healing and scar management.

## Figures and Tables

**Table 1:** Regulated genes in fetal banked cells compared to young banked cells.

Enter gene ID	Gene symbol	Gene name	Fold increase (expressed in log_2_)
3205	HOXA9	Homeobox A9	4.103
2890	GRIA1	Glutamate receptor, ionotropic, AMPA 1	4.050
3223	HOXC6	Homeobox C6	3.255
3202	HOXA5	Homeobox A5	3.243
2861	GPR37	G protein-coupled receptor 37 (Endothelin receptor type B-LIKE)	3.221
5789	PTPRS	Protein tyrosine phosphatase, receptor type, D	3.171
3206	HOXA10	Homeobox A10	3.084
2321	FLT1	FMS-related tyrosine kinase 1	3.060
9452	ITM2A	Integral membrane protein 2A	3.030
862	RUNX1T1	RUNT-related transcription factor 1; translocated TO, 1 (cyclin D-related)	2.985
1047	CLGN	Calmegin	2.916
744	MPPED2	Metallophosphoesterase domain containing 2	2.844
9056	SLC7A7	Solute carrier family 7, Member 7	2.806
1948	EFNB2	Ephrin-B2	2.770
23266	LPHN2	Latrophilin 2	2.714
9865	KIAA0644	KIAA0644 gene product	2.689
23705	CADM1	Immunoglobulin superfamily, member 4	2.607
1016	CDH18	Cadherin 18, type 2	2.553
2294	FOXF1	Forkhead box F1	2.543
140462	ASB9	DKFZP564L0862 protein	2.452
5947	RBP1	Retinol binding protein 1, cellular	2.394
83939	EIF2A	Eukaryotic translation initiation factor 2A, 65 KDA	2.276
5918	RARRES1	Retinoic acid receptor responder (tazarotene induced) 1	2.256
3306	HSPA2	Heat shock 70 KDA protein 2	2.211
5880	RAC2	RAS-related C3 botulinum toxin substrate 2 (RHO family, small GTP binding protein RAC2)	2.175
151230	KLHL23	Kelch-like 23	2.171
284	ANGPT1	Angiopoietin 1	2.171
2201	FBN2	Fibrillin 2 (congenital contractural arachnodactyly)	2.164
3880	KRT19	Keratin 19	2.163
57157	PHTF2	Putative homeodomain transcription factor 2	2.147
154796	AMOT	Angiomotin	2.121
1012	CDH13	Cadherin 13, H-cadherin (heart)	2.086
5507	PPP1R3C	Protein phosphatase 1, regulatory (inhibitor) subunit 3C	2.042
1908	EDN3	Endothelin 3	2.032
6319	SCD	Stearoyl-coa desaturase (delta-9-desaturase)	2.022
5307	PITX1	Paired-like homeodomain transcription factor 1	2.008
6899	TBX1	T-box 1	1.957
5099	PCDH7	BH-protocadherin (brain-heart)	1.939
3215	HOXB5	Homeobox B5	1.880
6664	SOX11	Sry (sex determining region Y)-box 11	1.862
4004	LMO1	Lim domain only 1 (rhombotin 1)	1.830
10924	SMPDL3A	Sphingomyelin phosphodiesterase, acid-like 3A	1.810
3233	HOXD4	Homeobox D4	1.810
3232	HOXD3	Homeobox D3	1.809
1305	COL13A1	Collagen, type XIII, alpha 1	1.772
444	ASPH	Aspartate beta-hydroxylase	1.767
5781	PTPN11	Protein tyrosine phosphatase, non-receptor type 11 (noonan syndrome 1)	1.749
4908	NTF3	Neurotrophin 3	1.741
7046	TGFBR1	Transforming growth factor, beta receptor I (activin a receptor type II-like kinase, 53 KDA)	1.722
5743	PTGS2	Prostaglandin-endoperoxide synthase 2 (prostaglandin G/H synthase and cyclooxygenase)	1.708
23475	QPRT	Quinolinate phosphoribosyltransferase	1.704
934	CD24	CD24 antigen	1.698
10773	ZBTB6	Zinc finger protein 482	1.685
2048	EPHB2	EPH receptor B2	1.624
23089	PEG10	Paternally expressed 10	1.617
26013	L3MBTL	Lethal (3) malignant brain tumor L(3)MBT protein (drosophila) homolog	1.614
3434	IFIT1	Interferon-induced protein with tetratricopeptide repeats 1	1.613
5156	PDGFRA	Platelet-derived growth factor receptor, alpha polypeptide	1.610
382	ARF6	ADP-ribosylation factor 6	1.610
2028	ENPEP	Glutamyl aminopeptidase (aminopeptidase A)	1.607
10643	IGF2BP3	Insulin-like growth factor 2 MRNA binding protein 3	1.604
8091	HMGA2	High mobility group at-hook 2	1.580
900	CCNG1	Cyclin G1	1.579
4192	MDK	Midkine (neurite growth-promoting factor 2)	1.577
552889	LOC552889	Hypothetical LOC552889	1.576
540	ATP7B	Atpase, CU++ transporting, beta polypeptide	1.565
2289	FKBP5	FK506 binding protein 5	1.543
4194	MDM4	MDM4, transformed 3t3 cell double minute 4	1.538
5764	PTN	Pleiotrophin	1.533
8935	SKAP2	SRC family associated phosphoprotein 2	1.528
273	AMPH	Amphiphysin	1.527
2799	GNS	Glucosamine (N-acetyl)-6-sulfatase (sanfilippo disease IIID)	1.519
5311	PKD2	Polycystic kidney disease 2 (autosomal dominant)	1.518
10402	ST3GAL6	ST3 beta-galactoside alpha-2,3-sialyltransferase 6	1.501
3764	KCNJ8	Potassium inwardly-rectifying channel, subfamily J, member 8	−1.501
6347	CCL2	Chemokine (C-C motif) ligand 2	−1.509
2030	SLC29A1	Solute carrier family 29	−1.517
2919	CXCL1	Chemokine (C-X-C MOTIF) ligand 1	−1.519
1839	HBEGF	Heparin-binding EGF-like growth factor	−1.522
3488	IGFBP5	Insulin-like growth factor binding protein 5	−1.539
5678	PSG9	Pregnancy specific beta-1-glycoprotein 9	−1.557
3643	INSR	Insulin receptor	−1.569
10974	C10ORF116	Chromosome 10 open reading frame 116	−1.580
4071	TM4SF1	Transmembrane 4 L six family member 1	−1.580
1832	DSP	Desmoplakin	−1.590
4487	MSX1	MSH homeobox homolog 1	−1.613
27295	PDLIM3	PDZ and LIM domain 3	−1.615
1294	COL7A1	Collagen, type VII, alpha 1	−1.617
182	JAG1	Jagged 1 (alagille syndrome)	−1.617
22891	ZNF365	Zinc finger protein 365	−1.619
4223	MEOX2	Mesenchyme homeobox 2	−1.660
8076	MFAP5	Microfibrillar associated protein 5	−1.665
51363	GALNAC4S-6ST	KIAA0598 gene product	−1.676
5354	PLP1	Proteolipid protein 1	−1.694
1410	CRYAB	Crystallin, alpha B	−1.699
4854	NOTCH3	Notch homolog 3	−1.743
116039	OSR2	ODD-skipped related 2	−1.753
10873	ME3	Malic enzyme 3, NADP(+)-dependent, mitochondrial	−1.760
5865	RAB3B	RAB3B, member RAS oncogene family	−1.764
10391	CORO2B	Coronin, actin binding protein, 2B	−1.770
22809	ATF5	Activating transcription factor 5	−1.782
6356	CCL11	Chemokine (C-C motif) ligand 11	−1.783
7869	SEMA3B	Semaphorin 3B	−1.802
8404	SPARCL1	SPARC-like 1 (MAST9, HEVIN)	−1.813
1004	CDH6	Cadherin 6, type 2, K-cadherin (fetal kidney)	−1.823
131578	LRRC15	Leucine rich repeat containing 15	−1.836
3855	KRT7	Keratin 7	−1.846
11145	HRASLS3	HRAS-like suppressor 3	−1.853
5919	RARRES2	Retinoic acid receptor responder	−1.862
5493	PPL	Periplakin	−1.882
27063	ANKRD1	Ankyrin repeat domain 1 (cardiac muscle)	−1.891
684	BST2	Bone marrow stromal cell antigen 2	−1.918
8190	MIA	Melanoma inhibitory activity	−1.932
7042	TGFB2	Transforming growth factor, beta 2	−1.950
6004	RGS16	Regulator of G-protein signalling 16	−1.969
7020	TFAP2A	Transcription factor AP-2 alpha	−2.008
3589	IL11	Interleukin 11	−2.022
8549	LGR5	Leucine-rich repeat-containing G protein-coupled receptor 5	−2.027
2277	FIGF	C-FOS induced growth factor	−2.029
4958	OMD	Osteomodulin	−2.047
10351	ABCA8	ATP-binding cassette, sub-family A (ABC1), member 8	−2.051
2065	ERBB3	V-ERB-B2 erythroblastic leukemia viral oncogene	−2.073
5649	RELN	Reelin	−2.130
6663	SOX10	SRY (sex determining region Y)-BOX 10	−2.172
2878	GPX3	Glutathione peroxidase 3 (plasma)	−2.183
1620	DBC1	Deleted in bladder cancer 1	−2.208
4629	MYH11	Myosin, heavy polypeptide 11, smooth muscle	−2.221
4046	LSP1	Lymphocyte-specific protein 1	−2.224
5730	PTGDS	Prostaglandin D2 synthase 21 KDA (BRAIN)	−2.252
6781	STC1	Stanniocalcin 1	−2.290
1675	CFD	Complement factor D (ADIPSIN)	−2.298
3490	IGFBP7	Insulin-like growth factor binding protein 7	−2.317
10439	OLFM1	Olfactomedin 1	−2.341
23090	ZNF423	Zinc finger protein 423	−2.372
1842	ECM2	Extracellular matrix protein 2	−2.426
11098	PRSS23	Protease, serine, 23	−2.431
7857	SCG2	SECRETOGRANIN II (CHROMOGRANIN C)	−2.448
2628	GATM	Glycine amidinotransferase	−2.463
358	AQP1	Aquaporin 1	−2.507
8418	CMAH	Cytidine monophosphate-N-acetylneuraminic acid hydroxylase	−2.533
2006	ELN	Elastin (supravalvular aortic stenosis, williams-beuren syndrome)	−2.589
3481	IGF2	Insulin-like growth factor 2 (somatomedin A)	−2.592
4804	NGFR	Nerve growth factor receptor (tnfr superfamily, member 16)	−2.625
2675	GFRA2	GDNF family receptor alpha 2	−2.646
249	ALPL	Alkaline phosphatase, liver/bone/kidney	−2.671
2596	GAP43	Growth associated protein 43	−2.678
11075	STMN2	Stathmin-like 2	−2.695
5803	PTPRZ1	Protein tyrosine phosphatase, receptor-type, Z polypeptide 1	−2.695
8744	TNFSF9	Tumor necrosis factor superfamily, member 9	−2.723
8644	AKR1C3	Aldo-keto reductase family 1, member C3	−2.837
8839	WISP2	WNT1 inducible signaling pathway protein 2	−2.910
124	ADH1C	Alcohol dehydrogenase 1A (CLASS I)	−2.927
1734	DIO2	Deiodinase, iodothyronine, type II	−2.947
1116	CHI3L1	Chitinase 3-like 1 (cartilage glycoprotein-39)	−3.329
5320	PLA2G2A	Phospholipase A2, group IIA (platelets, synovial fluid)	−3.355
3123	HLA-DRB6	Major histocompatibility complex, class II, DR beta 1	−3.415
3128	HLA-DRB5	Major histocompatibility complex, class II, DR beta 6	−3.415
4915	NTRK2	Neurotrophic tyrosine kinase, receptor, type 2	−3.552
3479	IGF1	Insulin-like growth factor 1 (somatomedin C)	−3.682
3897	L1CAM	L1 cell adhesion molecule	−3.706
347	APOD	Apolipoprotein D	−3.749
50486	G0S2	G0/G1switch 2	−3.913
2824	GPM6B	Glycoprotein M6B	−4.029
1299	COL9A3	Collagen, type IX, alpha 3	−4.076
9244	CRLF1	Cytokine receptor-like factor 1	−4.463
2662	GDF10	Growth differentiation factor 10	−4.813
1311	COMP	Cartilage oligomeric matrix protein	−4.840
